# Proteomic Mendelian randomization to identify protein biomarkers of telomere length

**DOI:** 10.1038/s41598-024-72281-7

**Published:** 2024-09-16

**Authors:** Jiaxuan Zhao, Kun Yang, Yunfei Lu, Linfeng Zhou, Haoran Fu, Jingbo Feng, Jinghua Wu

**Affiliations:** 1 Department of Clinical Laboratory, North China University of Science and Technology Affiliated Tangshan Maternal and Child Health Care Hospital, Tangshan, China; 2 Key Laboratory of Molecular Medicine for Abnormal Development and Related Diseases in Tangshan City, Tangshan, China; 3 The 982th Hospital of the People’s Liberation Army Joint Logistics Support Force, Tangshan, China

**Keywords:** Mendelian randomization, Telomere length, Protein, Senescence, Proteomics, Proteomics, Telomeres

## Abstract

Shortening of telomere length (TL) is correlated with many age-related disorders and is a hallmark of biological aging. This study used proteome-wide Mendelian randomization to identify the protein biomarkers associated with telomere length. Protein quantitative trait loci (pQTL) were derived from two studies, the deCODE Health study (4907 plasma proteins) and the UK Biobank Pharma Proteomics Project (2923 plasma proteins). Summary data from genome-wide association studies (GWAS) for TL were obtained from the UK Biobank (472,174 cases) and GWAS Catalog (418,401 cases). The association between proteins and TL was further assessed using colocalization and summary data-based Mendelian randomization (SMR) analyses. The protein–protein network, druggability assessment, and phenome-wide MR were used to further evaluate the potential biological effects, druggability, and safety of the target proteins. Proteome-wide MR analysis identified 22 plasma proteins that were causally associated with telomere length. Five of these proteins (APOE, SPRED2, MAX, RALY, and PSMB1) had the highest evidence of association with TL and should be prioritized. This study revealed telomere length-related protein biomarkers, providing new insights into the development of new treatment targets for chronic diseases and anti-aging intervention strategies.

## Introduction

Telomeres are located at the termini of every linear chromosome and are a specific type of DNA–protein complex. Telomeres and their associated proteins protect chromosomal ends to maintain genomic stability. During DNA replication, the ends of chromosomes cannot be completely replicated^1^, leading to the progressive shortening of telomeres with cell division and ultimately to cellular senescence^2^. Therefore, telomere length (TL) shortening has long been defined as a sign of senescence^3,4^. Several studies have demonstrated an association between shortened telomere length (often assessed using leukocyte TL) and human longevity and mortality^5,6^. Shortened telomeres have also been associated with an increased likelihood of age-associated diseases, including cardiovascular disease^7^, type 2 diabetes^8^, and some neurological disorders^9^.

Many factors affect TL, including lifestyle, nutrition, environmental exposure^10^, and genetics. For example, the relationship between TL and lifestyle factors (e.g., smoking and obesity) has been investigated^11,12^. TL is also affected by genetic variants [e.g., single nucleotide polymorphisms (SNPs)]^13^. It has also been shown that oxidative DNA damage can lead to telomere wear and tear or dysfunction, which can trigger cellular senescence and apoptosis^14^. Previous research has indicated that TL is also regulated by proteins such as WRAP53, which affects TL by regulating telomerase activity^15^, and other proteins that participate in cell cycle-related signaling pathways^16,17^. However, considering the limitation of the research scope, a large percentage of TL-related proteins have not yet been fully explored. Therefore, further insight into the molecular mechanisms of telomere maintenance is important for improving human health and developing therapeutic strategies against a number of age-related diseases.

Mendelian randomization (MR) analyses use genetic instrumental variables (IVs), specifically SNPs derived from genome-wide association studies (GWAS), to assess the causal impact of exposure on outcomes through genetic variation^18^. Based on the plasma protein data derived from two studies^19,20^, we first conducted a systematic study on the causal relationship between thousands of plasma proteins and TL using MR analysis. Sensitivity analyses and Steiger directionality tests were performed to exclude the effects of confounding factors and reverse causality. Subsequently, the level of association between the proteins and the TL was prioritized by integrating the results of repeatability MR, Bayesian colocalization, and summary data-based MR (SMR) analysis. In addition, the biological functions and potential interactions between proteins were identified through Gene Ontology enrichment analysis and protein–protein interaction (PPI) networks. Furthermore, we assessed the potential pleiotropy and side effects of the identified target proteins using a druggability evaluation and phenome-wide MR.

This study aimed to identify protein markers related to TL and provide new information for future research on potential therapeutic targets for chronic diseases to improve healthy aging.

## Methods

**Fig. 1:**
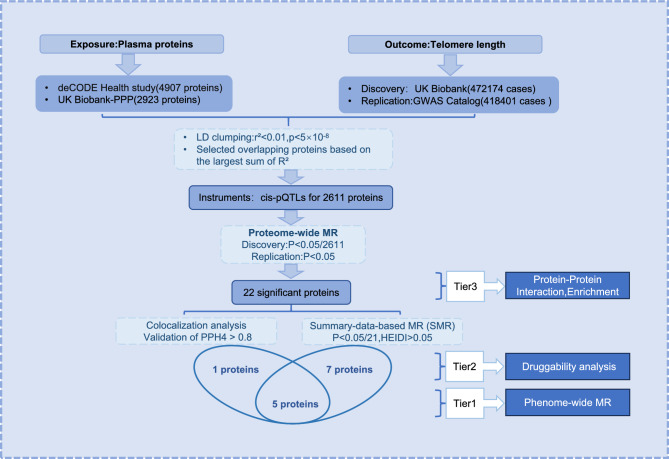
Overall framework of the study design.

The overall framework of the study design is shown in Fig. 1. This study used summary data derived from two proteomics studies to investigate the correlation between plasma proteins and TL using two-sample MR.

### Study population and datasets

#### Exposure data sources

We selected cis-protein quantitative loci (pQTL) from two GWAS datasets, the deCODE Health Study^20^ and the UK Biobank Pharma Proteomics Project (UKB-PPP)^19^, as genetic instrumental variables for plasma proteins. The UKB-PPP collected data on 2923 proteins via proteomic analysis of 54,219 plasma samples through the Olink platform. From these, we extracted cis-pQTL from 1314 plasma proteins as IVs for MR analysis. The deCODE Health study measured 4907 proteins in the plasma of 35,559 Icelanders using the SomaScan platform and found 18,084 correlated sequence variants between plasma protein levels. We extracted cis-SNPs from 1297 plasma proteins as IVs for the MR analysis. For plasma proteins that overlapped in the two studies, the protein exhibiting the highest total R^2^ was selected for retention.

#### Data sources of telomere length data

The main outcome data for this study were derived from the GWAS dataset, which is accessible to the public^21^. This dataset was a large-scale study that analyzed 20,134,421 SNPs and included 472,174 participants aged 40–69 years. The mean TL in the database was calculated using definitive quantitative PCR assays. A thorough quality check and technical adjustments were performed. Additionally, statistical adjustments were made for age to mitigate its impact on TL. The racial composition of the dataset was predominantly European Caucasians. The GWAS summary data from another TL study were obtained from Kessler et al. for replication^22^. This study quantified TL in 418,401 individuals of European ancestry using exome-wide sequencing.

### Proteomic MR analysis

In the MR analysis, we used summary GWAS statistics for plasma proteins as the exposure data and TL as the outcome data. We first selected SNPs that were strongly associated with proteins (*p* < 5 × 10^–8^) as IVs. Because the bias in the results may be caused by the high level of linkage disequilibrium (LD) between the instrumental variables, we set the LD between the selected SNPs to > 10,000 kb and the correlation coefficient (r^2^) < 0.01, to ensure mutual independence of each SNP. In addition, the robustness of the genetic IVs was estimated using R^2^ and F statistics (R^2^ = 2 × (1 − EAF) × EAF × beta^2^; F = R^2^ (N − 2)/(1 − R^2^))^23^ to increase power. The F-statistic for each IV in this study exceeded 10. A pQTL was considered a cis-pQTL if the lead SNP was located no more than one million base pairs from the transcription start site of the protein-encoding gene, and a pQTL found beyond this designated region was considered a trans-pQTL^24^. All genetic tools for plasma protein levels were constructed using cis-pQTLs, as the use of cis-pQTLs (pQTL close to protein-coding genes) is more conducive for adherence to the core assumptions of MR^25,26^. Information regarding IVs is provided in Supplementary Table S2.

The "TwoSampleMR" package with R language version 4.3.2 was used to perform the MR analysis. If two or more pQTL were available, the MR effect was estimated by inverse variance weighting. If only one pQTL was available, then the MR effect was estimated using the Wald ratio. To confirm the accuracy of the results, we performed sensitivity and heterogeneity tests based on Q statistics. Effect estimates were calculated using MR-Egger (adjusting for the residual correlation between variables), a model that accounts for horizontal pleiotropy. When heterogeneity was present, we chose the inverse variance-weighted (multiplicative random effects) model for the MR analysis. When pleiotropy was present, MR-Presso was used to test for outliers and confounders in the MR analysis. In the discovery cohort, we used the Bonferroni method for multiple correction of p-values, defining *p* < 1.91 × 10^–5^ (0.05/2611) as the significance level. In the validation cohort, we defined a nominal p-value < 0.05 as the level of significance. Finally, to examine whether a reverse causality bias existed, we performed a Steiger directionality test to determine whether our MR analyses were significantly affected by reverse causality.

### Bayesian colocalization analyses

We performed Bayesian colocalization analyses by using the "coloc" R package^27^ to evaluate whether consistent causal variation (rather than variation driven by linkage disequilibrium) was shared between TL and identified pQTLs. The colocalization analysis was based on five core hypotheses: H0, there was no causal variant for either of the two traits; H1, there was a causal variant for protein only; H2, there was a causal variant for TL only; H3, protein and TL had two different causal variants; and H4, protein and TL shared the same causal variant. Each of these hypotheses (H0, H1, H2, H3, and H4) corresponds to a posterior probability (i.e., PPH0, PPH1, PPH2, PPH3, and PPH4)^28^. We used SNPs within ± 1000 kb of the pQTL for pQTL-GWAS colocalization. If multiple pQTLs were present, a colocalization analysis was conducted separately for each pQTL, and the pQTL with the most robust colocalization evidence was displayed. The prior probabilities are respectively set at p1 = 1e−4, p2 = 1e−4, and p12 = 1e−5. We considered that PPH4 (posterior probability that protein and TL shared the same causal variant) greater than 80% constitutes robust evidence in favor of colocalization^29,30^.

### Summary-data-based Mendelian randomization (SMR)

SMR analysis was used as a complementary method to further explore the causal relationship between MR-identified proteins and TL. SMR analysis was conducted using SMR software version 1.3.1. The heterogeneity in the dependent instrument (HEIDI) test was also applied. When PHEIDI > 0.05, the link between protein and TL was not influenced by LD. The Bonferroni correction was applied to adjust the results of multiple testing, setting the threshold of significance for SMR at a p-value of < 2.38 × 10^−3^ (21 target proteins). Causal effects were considered to be statistically significant and not driven by cascading imbalances when the p-value was < 2.38 × 10^−3^ and P-HEIDI > 0.05.

We categorized the MR-identified proteins of the discovery cohort into three tiers based on results of association with TL. Proteins that passed both the colocalization analysis (PPH4 > 80%) and the SMR analysis (*p* < 2.38 × 10^−3^) and HEIDI test (*p* > 0.05) were defined as Tier 1. Proteins that passed only the colocalization analysis or the HEIDI test were defined as Tier 2. Proteins failed both the colocalization and HEIDI test due to insufficient statistical power or missing data were defined as Tier 3.

### PPI network, enrichment, and druggability assessment

We constructed a PPI network using the STRING database and Cytoscape software for visual representation to probe potential interactions between TL-associated proteins. Biological functions of TL-associated proteins were further investigated by Gene Ontology (GO) enrichment analysis, and the results were visualized using R packages such as "clusterProfiler" and "pathview"^31^. In addition, to explore whether the aforementioned proteins can be used as targets of existing drugs or druggable gene targets, we searched for interactions between these proteins and drugs and further explored the druggability of these proteins using the DrugBank^32^ database.

### Phenome-wide MR analysis (MR-PheWAS)

To assess the horizontal pleiotropy and possible side effects of potential target proteins more comprehensively, we performed MR-PheWAS analyses of the Tier 1 proteins as exposures. The outcome data for conducting PheWAS were obtained from the UK Biobank, which tested 28 million SNPs for 1403 disease phenotypes in 408,961 White British participants using the SAIGE platform (https://www.leelabsg.org/resources)^33^. Considering that disease phenotypes with a sample size of more than 500 cases have higher representativeness and stronger statistical validity, we subjected 783 disease phenotypes obtained from selection (n > 500) (Supplementary Table 8) to MR-PheWAS analyses^34^. More detailed information is provided in a previous publication^33^. Bonferroni correction was applied to the p-value to adjust for multiple tests, setting the threshold of significance at *p* < 0.05/783.

## Results

### Proteomic MR identified 22 plasma proteins associated with TL

**Fig. 2:**
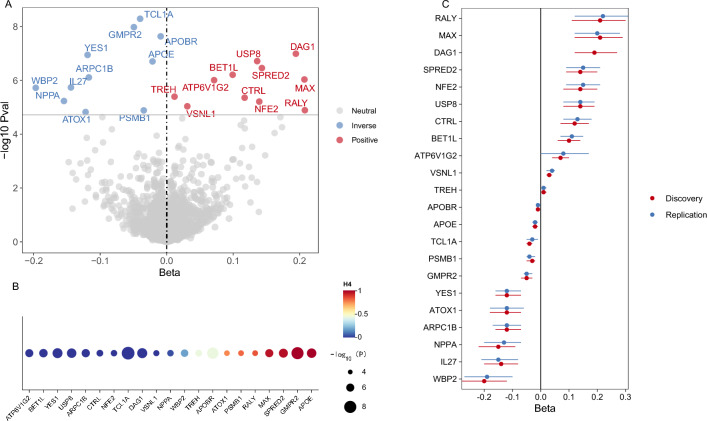
Summary data from the two-stage MR (discovery and replication) and colocalization analysis on the causal relationship between proteins and TL. (**A**) Volcano plot of the MR results in the discovery stage between 22 plasma proteins and TL. The tagged proteins passed multiple test corrections (*p* < 1.91 × 10^–5^). (**B**) Bubble plot showing the results of colocalization analysis between plasma proteins and TL. (**C**) MR analysis of 22 proteins in the discovery and replication datasets.

Proteomic MR analysis at the discovery stage revealed that 22 proteins from the two protein databases were significantly associated with TL (*p* < 1.91 × 10^–5^) after Bonferroni correction (Supplementary Table S3, Fig. 2A). Specifically, we observed 11 proteins for which increased protein abundance was significantly and positively associated with TL, including DAG1, USP8, SPRED2, BET1L, MAX, ATP6V1G2, TREH, CTRL, NFE2, VSNL1, and RALY. Increased protein abundance of 11 proteins was significantly negatively correlated with TL, including in PSMB1, ARPC1B, WBP2, GMPR2, NPPA, APOBR, APOE, ATOX1, IL27, and TCL1A, and YES1.

These associations were generally consistent in direction in other analyses including maximum likelihood, weighted median, and MR-Egger. No pleiotropy was observed (p > 0.05). For the two proteins (e.g., APOE and TREH) where heterogeneity (P_heterogeneity_ < 0.05) was present, the results of inverse variance weighted (IVW; multiplicative random) effects were used. The F-statistics for each IV exceeded 10, indicating robustness (Supplementary Table S3). In addition, the Steiger directionality test revealed a true causal relationship between plasma proteins and TL (Supplementary Table S3).

In the replication stage of the study, 20 proteins were validated in the GWAS catalog database based on the Wald or IVW methods (*p* < 0.05) (Table 1, Fig. 2C, Supplementary Table S4), and the IV for DAG1 could not be detected owing to heterogeneity in the outcome data.

**Table 1:** Summary data of MR, colocalization analysis, and SMR for the 22 MR-identified proteins.

Protein	MR			Colocalization PPH4	SMR			Tier
	*P*_*discovery*_	*P*_*replication*_	Beta		Beta	*P*	*P*_HEIDI_	
APOE	1.99e−07	1.80e−05	− 0.02	1.00	− 0.02	2.51e−10	0.06	1
SPRED2	3.50e−07	2.47e−07	0.14	0.97	0.14	7.66e−06	0.17	1
MAX	9.31e−07	9.40e−07	0.21	0.96	0.11	2.46e−14	0.16	1
RALY	1.29e−05	1.63e−05	0.21	0.84	0.13	4.46e−05	0.77	1
PSMB1	1.31e−05	8.65e−07	− 0.03	0.81	− 0.03	4.34e−05	0.29	1
GMPR2	1.06e−08	1.37e−07	− 0.05	1.00	− 0.05	1.12e−05	3.71e−03	2
ATOX1	1.51e−05	4.44e−05	− 0.12	0.76	− 0.12	8.45e−05	0.57	2
APOBR	2.33e−08	4.08e−05	− 0.01	0.44	− 0.01	8.97e−05	0.12	2
WBP2	1.90e−06	1.38e−05	− 0.20	0.18	− 0.20	1.28e−04	0.36	2
NPPA	5.86e−06	1.20e−04	− 0.15	0.04	− 0.15	1.49e−04	0.40	2
DAG1	1.03e−07	–	0.19	5.65e−06	0.19	1.73e−05	0.61	2
ARPC1B	7.80e−07	1.44e−06	− 0.12	3.81e−12	− 0.12	2.94e−05	0.09	2
USP8	1.94e−07	5.97e−07	0.14	9.84e−17	0.14	5.49e−06	0.14	2
IL27	1.84e−06	3.25e−06	− 0.14	–	–	–	–	3
TREH	4.04e−06	3.43e−07	0.01	0.44	0.01	8.89e−05	0.04	3
VSNL1	9.22e−06	1.40e−06	0.03	1.85e−05	0.03	2.80e−08	0.02	3
TCL1A	5.17e−09	2.75e−04	− 0.04	7.39e−08	− 0.03	2.91e−09	1.09e−04	3
NFE2	6.17e−06	3.41e−06	0.14	2.21e−08	0.14	7.11e−06	1.38e−06	3
CTRL	4.42e−06	1.39e−06	0.12	7.69e−11	0.11	1.51e−04	0.01	3
YES1	1.14e−07	5.58e−07	− 0.12	4.27e−17	− 0.14	4.34e−06	9.99e−06	3
BET1L	6.22e−07	1.20e−07	0.10	3.86e−20	0.10	2.85e−08	3.43e−08	3
ATP6V1G2	9.89e−07	0.06	0.07	2.09e−30	0.07	3.81e−17	5.60e−04	3

PPH4 means the posterior probability that protein and TL shared the same causal variant. Tier 1 means the MR-identified proteins passed both the colocalization (PPH4 > 0.8) and the SMR analysis (*P* < 2.38 × 10 ^-3^) and HEIDI test (*P*_*HEIDI*_ > 0.05); Tier 2 means the MR-identified proteins passed only the colocalization or the HEIDI test; Tier 3 means the MR-identified proteins failed both the colocalization and HEIDI test.

### Colocalization analysis identified 6 proteins sharing genetic variation with TL

To identify proteins and TL driven by common LD variants, we performed co-localization analyses (Supplementary Table S5) on the 21 protein-TL pairs from the aforementioned results (IL27 lacked complete GWAS data and could not be examined) and identified six proteins (e.g., APOE, SPRED2, MAX, RALY, PSMB1, and GMPR2) that showed strong evidence of co-localization with TL (PP.H4 > 0.8) (Table 1, Supplementary Table S5, and Fig. 2B).

### SMR identified 12 proteins associated with TL

To further validate the obtained results, we performed SMR and HEIDI tests on the 21 proteins with complete GWAS summary data with the TL of the aforementioned discovery stage. All 21 proteins passed the SMR test (*p* < 2.38 × 10^−3^) after Bonferroni correction, and 12 of them passed the HEIDI test (*p* > 0.05) (Table 1, Supplementary Table S6).

### Summary findings

Combining these findings, we categorized evidence for the association of these proteins with TL into three tiers (Table 1). Five proteins (e.g., APOE, SPRED2, MAX, RALY, and PSMB1) that passed both the colocalization analysis as well as the SMR analysis and HEIDI test based on multiple test correction by MR analysis were categorized as Tier 1. MR-significant proteins (e.g., GMPR2, ATOX1, APOBR, WBP2, NPPA, DAG1, ARPC1B, and USP8) that passed only the colocalization analysis or the HEIDI test were categorized as Tier 2. Proteins that passed the MR analysis but failed both the colocalization and SMR analyses because of insufficient statistical power or missing data (e.g., TREH, VSNL1, TCL1A, NFE2, CTRL, YES1, BET1L, and ATP6V1G2) were categorized as Tier 3.

### PPI, enrichment, and evaluation of druggability

**Fig. 3:**
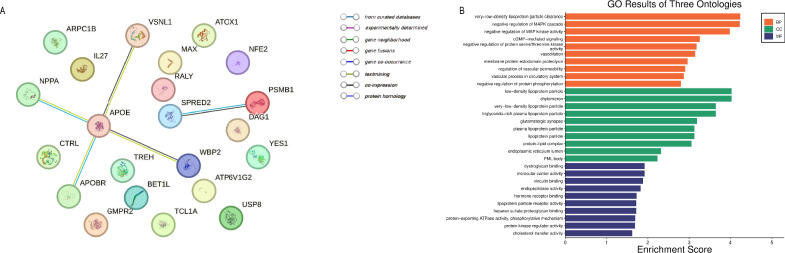
PPI network and GO enrichment analysis of 22 proteins from discovery stage MR. (**A**) PPI network of proteins associated with TL. (**B**) GO enrichment of proteins associated with TL. *BP* biological processes, *MF* molecular functions, *CC* cellular components.

To deepen the understanding of the potential biological relationships of these proteins affecting TL and their functions, we constructed a PPI network on the significant proteins analyzed by MR in the discovery stage (Fig. 3A). We observed potential interactions between APOE and VSNL1, NPPA, WBP2, and APOBR. GO enrichment results showed that multiple biological processes may affect TL, including very-low-density lipoprotein particle clearance, dystroglycan binding, and negative regulation of the MAPK cascade (Fig. 3B).

Subsequently, we assessed the druggability of the 13 MR-significant proteins analyzed by colocalization or SMR as previously described (Tier 2). DrugBank found that four of these proteins (e.g., PSMB1, ATOX1, APOE, and ARPC1B) were targets for drug development (Supplementary Table S7). For example, some drugs targeting PSMB1 (e.g., carfilzomib and bortezomib) have received approval as proteasome inhibitors for managing individuals diagnosed with relapsed or resistant multiple myeloma. Drugs targeting ATOX1 (e.g., cisplatin) have been approved for the treatment of metastatic ovarian tumors, metastatic testicular tumors, and advanced bladder cancer. Benzamidine is used to treat oral pain and inflammation.

### Phenome-wide MR analysis of Tier 1 proteins

**Fig. 4:**
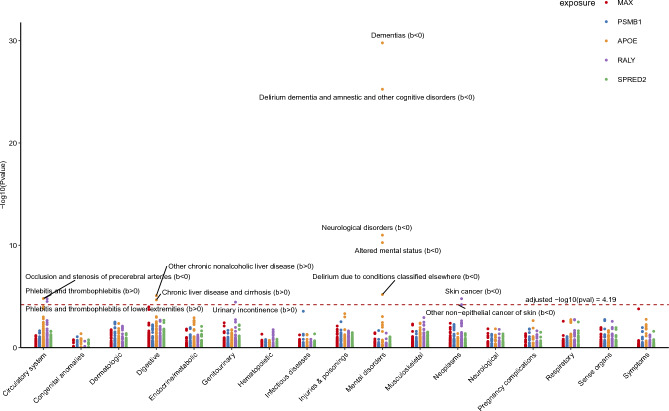
Manhattan plot for MR-PheWAS results of five proteins in Tier 1. Each dot symbolizes a disease trait, and different colors represent MR results for different protein expressions.

The five proteins of Tier 1 passed all tests and had the highest level of evidence of association with TL. Thus, we evaluated their pleiotropic properties and safety as potential protein targets. Through a more extensive MR analysis of 783 phenotypes, we found that higher APOE levels were positively associated with the risk of chronic liver disease and cirrhosis (β = 0.17, *p* = 2.16e−05) but negatively associated with the risk of dementia and other cognitive disorders (β = − 0.76, *p* = 5.58e−26). Moreover, RALY was positively associated with the risk of phlebitis and thrombophlebitis (β = 2.36, *p* = 1.67e−05). No other phenotypes were significantly associated with MAX, PSMB1, or SPRED2 (*p* < 0.05/783) (Fig. 4). The summary data are presented in Supplementary Tables S8–S13.

## Discussion

To our knowledge, this is the first MR study to comprehensively explore the effects of plasma proteome-centric targets of specific proteins on TL. This MR study analyzed the association between plasma proteins and TL, supplemented by colocalization analysis and SMR analyses. In the discovery stage of the proteomic MR study, 22 protein markers associated with TL were identified, of which elevated levels of 11 proteins and reduced levels of another 11 correlated with increased TL. Replication MR validated 20 of 22 candidate proteins. Among these, five proteins (e.g., APOE, SPRED2, MAX, RALY, and PSMB1), were strongly supported by colocalization and passed the SMR and HEIDI tests, with the highest level of evidence of association with TL. Among these, an increased abundance of SPRED2, MAX, and RALY proteins was positively associated with TL, whereas an increased abundance of APOE and PSMB1 proteins was negatively associated with TL. Phenome-wide MR analyses indicated potential target effects, both favorable and unfavorable, and highlighted safety issues regarding the use of target proteins for medicinal purposes. Overall, these findings provided new insights into the biological mechanisms and potential effector targets of TL.

Among the candidate proteins identified, some have been suggested to be associated with TL in terms of gene polymorphisms or protein levels. For example, previous GWAS have shown that genetic polymorphisms in *APOE, NFE2, SPRED2, MAX, and TCL1A* are associated with TL^21,35,36^, suggesting that the data sources used in the current analysis have good validity. Among them, APOE, SPRED2, and MAX were classified as Tier 1 proteins with the highest level of evidence in this study. Recently, it has been shown that APOE may indirectly regulate telomere length in neural stem cells (NSCs) by inhibiting sirtuin 1(SIRT1) and fibroblast growth factor 2 (FGF2)^37^. Dhillon et al*.* showed that Apolipoprotein-ε4 (*APOE-ε4*) carriers have shorter telomeres than non-carriers and that carriers suffer from related diseases such as dementia^38^. However, we found that the two most common variants of *APOE* subtypes, rs429358 and rs7412, did not appear in our study, which may stem from differences in assay methodology, population structure, and genetic background from previous studies. Interestingly, the SNP rs483082 in this study and *APOE-ε4* (encoded by rs429358) signaling showed a strong association in previous studies and together affect Alzheimer's disease-related pathological features, especially in regulating Aβ40/42 levels^39,40^. This may be due to their location in the same linkage disequilibrium (LD) region. RALY is an RNA-binding protein^41^ that has been found to be a component of the Telomeric repeat-containing RNA(TERRA) interactome in mouse embryonic stem cells^42^. TERRA is a long-stranded, non-coding RNA transcribed from telomeres that plays a key role in maintaining telomere length and stability^43^. Recent studies have shown that RALY depletion leads to reduced levels of TERRA, disrupting its localization at telomeres and eventually causing telomere damage^44^. These findings are consistent with the results of our study. Our study further confirmed the causal association of these genes with TL at the protein level and extended the evidence for the genetic association of downstream mechanisms.

Our study identified several novel TL protein biomarkers. For example, the proteasome subunit beta type-1 (PSMB1) has the strongest evidence (Tier 1). Our study suggests that PSMB1 has a potential inhibitory effect on TL. However, no evidence on the association between PSMB1 and TL has been obtained from observational epidemiological and experimental studies. PSMB1 is the β6 subunit in the 20S proteasome and serves as a checkpoint for the assembly of proteasome dimerization^45,46^. Moreover, PSMB1 plays a critical role in maintaining protein homeostasis and is involved in various biological processes. Previous studies have shown that PSMB1 promotes proteasome-dependent degradation of IκB kinase ε (IKK-ε) and inhibits interferon signaling^47^. Recently, new evidence suggests that PSMB1 acts as a negative regulator of the Rankl-induced NF-κB pathway, targeting IKK-β to inhibit NF-κB activation^48^. There is evidence for an association between NF-κB activation and telomerase reverse transcriptase (*TERT*) expression. For example, transcriptional up-regulation of *TERT* was selectively induced by activation of NF-κB in endosomal smooth muscle cells^49^, and it was observed in mouse tissues that NF-κB may contribute to the activation of *TERT* expression^50^, which is essential for the maintenance of TL^50^. Therefore, it is reasonable to hypothesize that PSMB1 may reduce *TERT* expression by targeting the inhibition of NF-κB activity, ultimately leading to telomere length shortening. However, further epidemiological and experimental studies are required to confirm these findings.

One advantage of our study is that it is the first large-scale study to jointly apply three methods (MR, colocalization, and SMR) to investigate the causal effects between TL and protein biomarkers. The MR design reduces the bias from confounding factors and reverses causality, thereby enhancing the reliability of the causal inference. Additionally, the colocalization analysis has been shown to be an effective method for reducing biases caused by potential LD. The large sample size of the GWAS significantly enhanced the statistical robustness of our analysis. In addition, the consistency of the MR results between the discovery and replication datasets for TL provides strong evidence supporting our conclusions. Our study had some limitations. First, we only included cis-pQTL, which is closer to the protein-coding gene, as a genetic IV for the MR analysis. Therefore, we cannot exclude other proteins that have significant trans-pQTL but were not included in the analysis due to the screening conditions. Second, strict evidence grading standards and significance threshold screening methods may underestimate the persuasive power of certain TL-associated proteins, resulting in false negative results. For example, the absence of complete summary data precluded the possibility of conducting colocalization and SMR analyses on IL27. Furthermore, because our GWAS dataset exclusively included European populations, the applicability of our findings to different ethnicities and groups may be limited^51^. Therefore, a larger cross-race meta-analysis is required to extend the identification of TL-associated causal variants to other ethnic groups. Finally, MR only provides insights into causal associations and its direction, because genetic variation reflects the lifelong effects of changes in protein levels on TL rather than making quantitative estimates^52^. Therefore, future studies are necessary to further validate the relationship between identified proteins and TL and to clarify the potential biological mechanisms through population-based studies as well as in vivo and in vitro experiments.

## Conclusions

In this study, we identified plasma proteins associated with TL through comprehensive MR analysis, and these telomere-associated genetic determinants represent potential therapeutic targets for aging-related diseases and deepen the understanding of the mechanisms of telomere biology and its relationship to aging.

## Supplementary Information

Supplementary Information.

## Data Availability

All GWAS data used in this study are publicly available, and the GWAS summary data for TL are available from the MRC-IEU OpenGWAS database (https://gwas.mrcieu.aco/) and GWAS Catalog (https://www.ebi.ac.uk/gwas/). Summary data for plasma proteins are available from UKB-PPP (https://www.ukbiobank.ac) and deCODE (https://www.decode.com/summarydata/).
